# Low-Cost 3D-Printed Kaolin–Rice-Husk Microporous Membranes for Microplastic Removal: A Comparative Study with a Commercial Ceramic Membrane

**DOI:** 10.3390/membranes16100346

**Published:** 2026-10-19

**Authors:** Gulzat Demeuova, Aliya Satayeva, Zhanserik Shadiyar, Alzhan Baimenov, Elizabeth Arkhangelsky, Zhuldyz Baimenova, Arailym Kamal

**Affiliations:** 1 School of Engineering, Nazarbayev University, Astana 010000, Kazakhstan; gdemeuova@nu.edu.kz (G.D.); zhanserik.shadiyar@nu.edu.kz (Z.S.);; 2 Laboratory of Engineering Profile, Satbayev University, Almaty 050000, Kazakhstan; aliya.satayeva@nu.edu.kz (A.S.); alzhan.baimenov@satbayev.university (A.B.); zhuldyz.baimenova@gmail.com (Z.B.)

**Keywords:** microplastics, polyethylene (PE), polyethylene terephthalate (PET), 3D printing, ceramic membranes, rice husk, water filtration

## Abstract

Microplastics (MPs) are pervasive emerging contaminants in freshwater and drinking-water systems, and conventional treatment processes may achieve insufficient removal of smaller MP fractions. Membrane filtration provides an effective barrier to MPs; however, commercial ceramic membranes can be relatively expensive. In this work, low-cost porous ceramic membranes were fabricated by extrusion-based three-dimensional (3D) printing of a kaolin–rice husk paste, followed by sintering at 1200 °C. The removal of model spherical polyethylene (PE) and irregularly shaped polyethylene terephthalate (PET) MPs across different particle-size fractions was evaluated using dead-end filtration and benchmarked against a commercial ceramic membrane. The 3D-printed membrane removed 58.3 ± 3.6% of 1–10 µm PE particles, while complete removal was achieved for the 75–90, 300–355, and 600–710 µm PE fractions. For PET, removal efficiencies of 80.8 ± 0.9%, 94.9 ± 1.8%, and 100% were obtained for the 10–45, 45–75, and 75–150 µm fractions, respectively. By comparison, the commercial membrane achieved 100% removal across all PE and PET fractions tested. The fabricated membrane had a modal pore size of 0.80 µm and an estimated cost of USD 2.98, compared with USD 54.45 for the commercial 0.45 µm membrane. Thus, the 3D-printed membrane effectively removed medium and large MP fractions, while its estimated fabrication cost was approximately 94.5% lower than the purchase cost of the commercial membrane used for comparison. Although its larger pore size resulted in incomplete removal of the smallest MP fractions, the combination of effective removal of larger MPs and substantially lower estimated fabrication cost demonstrates the potential of extrusion-based 3D printing as an alternative approach for producing ceramic membranes for MP removal.

## 1. Introduction

Synthetic polymers underpin modern manufacturing, and their production continues to climb: global plastic output reached approximately 430.9 million tons in 2024, of which packaging constitutes the largest end-use sector [1]. Polyethylene terephthalate (PET), a semi-crystalline thermoplastic polyester, is among the most heavily produced packaging polymers and dominates single-use beverage bottles. The chemical robustness that makes PET desirable also renders it environmentally persistent: weathering and mechanical abrasion slowly break discarded PET into microplastics (MPs) and, ultimately, nanoplastics. According to ISO 24187:2023 [2], MPs are solid, water-insoluble plastic particles with a longest dimension between 1 µm and 1 mm, and large MPs are those between 1 mm and 5 mm; particles smaller than 1 µm are conventionally referred to as nanoplastics [3]. Such polymeric fragments now pervade aquatic environments [4] and have been detected within the human body [5], including in blood, where PET is among the polymers most frequently identified [6,7], and in brain tissue [8], intensifying concern over the consequences of long-term exposure. In service, plastics such as fishing nets, agricultural mulch films, and packaging are additionally subjected to mechanical stresses, including tension, friction, and water impact, which can drive the release of polymer micropollutants into water through stress-induced phase separation [9]. Drinking water is a major exposure pathway, and PET is a recurrent component of the MP burden, in part because its widespread use in packaging contributes to particle shedding into bottled water [10]. As chemically inert polymeric particles that resist ready degradation, PET MPs must generally be eliminated by physical separation, making their removal from water a pressing treatment objective.

Among the available separation strategies, including coagulation [11], adsorption [12], and biological treatment, membrane filtration is widely regarded as the most effective and reliable approach for MP removal [13]. Microfiltration (MF), ultrafiltration (UF), nanofiltration (NF), and reverse osmosis (RO) retain upward of 95% of microplastics by size exclusion, and ceramic MF applied as a tertiary step has been shown to provide more than 72% additional removal of residual MPs, raising overall process efficiency above 96% [14]. MP removal is predominantly conducted by polymeric membranes, such as poly(vinylidene fluoride) and polyethersulfone [15,16]. Polymeric membranes have two notable limitations: their susceptibility to fouling and their relatively limited chemical and thermal stability. More importantly, in the context of microplastic removal, oxidative or chemical cleaning may promote the release of polymer fragments from the membrane into the permeate, potentially introducing a secondary source of MP contamination [17]. Inorganic ceramic membranes avoid this secondary release, are more hydrophilic, and resist flux decline more effectively than their polymeric counterparts [3]. Their principal drawback is cost, as conventional alumina- and zirconia-based ceramics remain expensive to fabricate, with reported prices on the order of USD 500–3000 per square meter [18].

This economic barrier has motivated the development of ceramic membranes made from low-cost, waste-derived precursors. Rice husk is an abundant agricultural residue and a rich silica source, whereas kaolin is an inexpensive, widely available clay. Membranes derived from such precursors combine adequate mechanical strength with high porosity and flux; rice-husk-derived silica ceramic membranes, for example, have achieved approximately 50% porosity, ~71 MPa mechanical strength, and pure water flux near 300 L m^−2^ h^−1^ at a sintering temperature of 1200 °C [19], while kaolin-based agro-waste ceramic membranes have been demonstrated for water-treatment duties such as bacterial removal [20]. Low-cost clay and agro-waste ceramic membranes have already been applied to microplastic removal, including composite membranes that retain mixtures of PET, polyethylene (PE), and polystyrene from wastewater [21]. The rice-husk–kaolin precursor system and its use for microplastic filtration are therefore established rather than novel in themselves.

The conventional methods of ceramic membrane fabrication include uniaxial pressing, slip and tape casting, and extrusion—which constrain geometry and afford limited control over pore architecture [22]. Additive manufacturing (3D printing) removes these constraints, enabling tailored geometries, controlled and graded porosity, and reproducible complex structures that cannot be obtained by conventional shaping methods such as knife casting; despite this potential, its application to membrane-based water treatment remains limited [23].

In this study, a rice-husk–kaolin ceramic membrane was produced by 3D printing and evaluated for the removal of microplastics from water. The ceramic membrane is fabricated entirely from abundant agricultural and mineral wastes, offering a key economic advantage. Its performance was benchmarked against that of a commercial TAMI ceramic (TAMI Industries, Nyons, France) UF membrane to determine whether comparable PET microplastic rejection can be achieved at a substantially lower fabrication cost.

## 2. Materials and Methods

### 2.1. Membrane and Materials

Commercial TAMI ceramic membranes with a 0.45 μm pore size were purchased from Sterlitech, USA. Kaolin and rice husk (RH) were purchased from Chemistry and Technology Co., Ltd. (Almaty, Kazakhstan), and used as the ceramic precursor and pore former, respectively. Ethanol (≥96.0%; Sigma-Aldrich, St. Louis, MO, USA) was used as a dispersant for selected PE suspensions. All aqueous solutions used in membrane fabrication, filtration and analytical characterization experiments were prepared using deionized water (DI) (MilliQ, Burlington, MA, USA). Spherical PE microspheres (*p* = 0.96 g/cm^3^) were supplied by Cospheric LLC (Santa Barbara, CA, USA), and irregularly shaped PET fragments (*p* = 1.40 g/cm^3^) were purchased from Chemical Plastic Raw Materials, Jinhua, China, and fractionated using a stack of test sieves supplied by Thermo Fisher Scientific (Endecotts Ltd., London, UK) with nominal apertures of 10, 45, 75, and 150 µm—the 10 µm sieve being of the electroformed type—to yield three size fractions: 10–45, 45–75, and 75–150 µm. Each fraction comprised the particles passing through the coarser sieve and retained on the next finer one.

### 2.2. Fabrication of the 3D-Printed Ceramic Membranes

**Figure 1:**
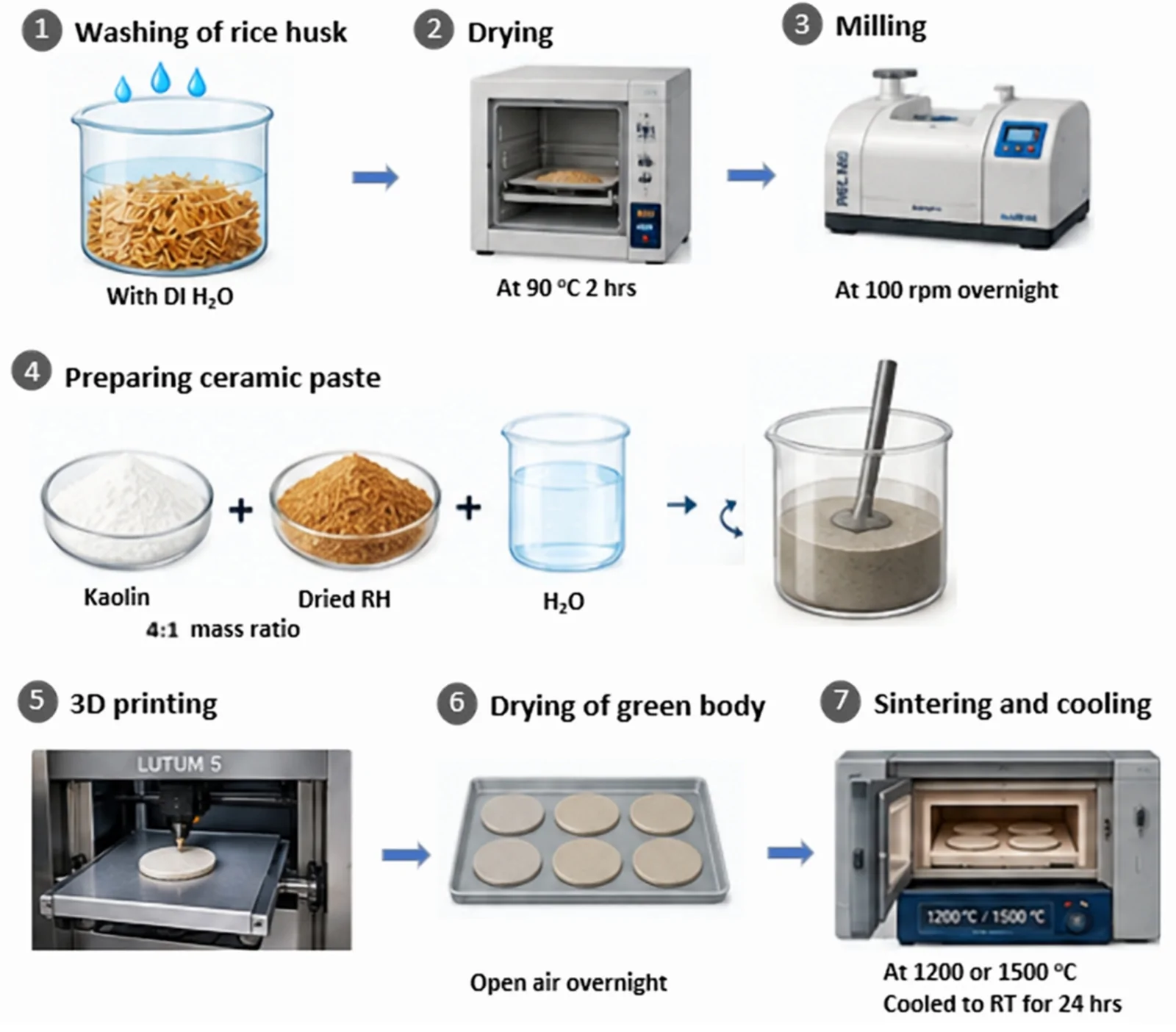
Schematic illustration of the fabrication of the 3D-printed kaolin–rice-husk ceramic membrane.

The methodology employed for the membrane fabrication was similar to that of Bartoletti et al. [24], with the exception of the feedstock materials. RH was first washed with DI water to remove dust and impurities. The cleaned RH was dried in an oven at 90 °C for 2 h, cooled to room temperature and milled in a PM 100 ball mill (Retsch, Haan, Germany) at 100 rpm for six 6-h cycles each to obtain a fine powder. A printable ceramic paste was prepared by combining kaolin and rice-husk powder at a kaolin mass ratio of 4:1 and mixing the mixture with water. The prepared paste was homogenized and hand-rolled on a flat surface to a workable consistency and then loaded into the 3D printer. Disc-shaped membranes were fabricated using extrusion-based 3D printing with a LUTUM 3D printer (VormVrij 3D, Afferden, The Netherlands). Therinted ceramic membranes were dried, and then sintered in a muffle furnace (LHT 04/17, Nabertherm, Lilienthal, Germany) at temperatures of 1200 °C and 1500 °C, followed by cooling to room temperature over 24 h. The fabrication process of the 3D-printed kaolin–rice-husk ceramic membranes is presented in Figure 1 and the process parameters are listed in the Supplementary Materials (Table S5).

### 2.3. Characterization of the 3D-Printed and Commercial Membranes

The functional groups of the prepared membranes were assessed using an Attenuated Total Reflectance Fourier Transform Infrared (ATR-FTIR) spectrometer (Nicolet Summit X, Thermo Scientific, Madison, WI, USA). The samples were placed directly onto a diamond ATR crystal and scanned from 4000 to 400 cm^−1^ at a resolution of 4 cm^−1^, and 32 scans were averaged for each spectrum. Background spectrum were collected prior to each analysis and the ATR crystal was cleaned with ethanol between measurements to avoid cross-contamination.

The surface morphology and elemental distribution of the membranes were examined using a Crossbeam 540 FIB-SEM scanning electron microscope (SEM) (Carl Zeiss Microscopy GmbH, Jena, Germany) equipped with an energy-dispersive X-ray spectroscopy (EDS) detector. SEM images were acquired at accelerating voltages of 5–20 kV. Prior to SEM analysis, the samples were sputter-coated with an approximately 10 nm thick gold layer to minimize surface charging. EDS analysis was subsequently performed to determine the elemental composition and distribution of the constituent elements. The open porosity, modal pore diameter, and pore-size distribution of the 3D-printed membranes were characterized using mercury intrusion porosimetry (MIP). The measurements were performed using a DHP 200 high-pressure unit (Stratum, Stavanger, Norway) and an AutoPore IV porosimeter (Micromeritics, Norcross, GA, USA), which determined pore characteristics by forcing mercury into the membrane pores under controlled pressure.

The membrane surface wettability was evaluated by measuring the water contact angle using an OCA 25 instrument (DataPhysics Instruments GmbH, Filderstadt, Germany) following the standard sessile-drop method. A membrane specimen was positioned on the instrument stage, and a 5 µL droplet of deionized (DI) water was carefully placed on its surface. The contact angle was subsequently calculated from the droplet profile using the instrument’s image analysis software.

Thermogravimetric analysis (TGA) of the samples was conducted using a NETZSCH STA 449 F3 Jupiter (NETZSCH-Gerätebau GmbH, Selb, Germany) simultaneous thermal analyzer. Approximately 5.8–18.05 mg of each sample was placed in an Al_2_O_3_ crucible and heated from 30 to 1300 °C at a heating rate of 5 °C min^−1^ under an argon atmosphere. Al_2_O_3_ was used as the reference compound‚ and the thermal stability and decomposition behavior of the samples were determined by the data obtained from the thermogravimetric curves.

### 2.4. Membrane Filtration Experiments

Model MP suspensions were prepared in DI water using PE particles in four nominal size ranges (1–10, 75–90, 300–355, and 600–710 µm) and PET particles in three ranges (10–45, 45–75 and 75–150 µm), at concentrations of 10 and 100 mg/L. All tested fractions fall within the ISO 24187:2023 microplastic range (1 µm–1 mm). PE is hydrophobic, has a lower density than water, and tends to aggregate in water, PE feed solutions were prepared in a 70% ethanol–DI solution. According to Fang et al. [25], a 70% ethanol solution provides optimal conditions for both the suspension of plastic particles and the reduction in PE aggregation. PET suspensions were prepared in water without ethanol. The initial particle-size distribution (PSD) of each feed was measured before filtration and compared with that of the permeate to quantify removal.

**Figure 2:**
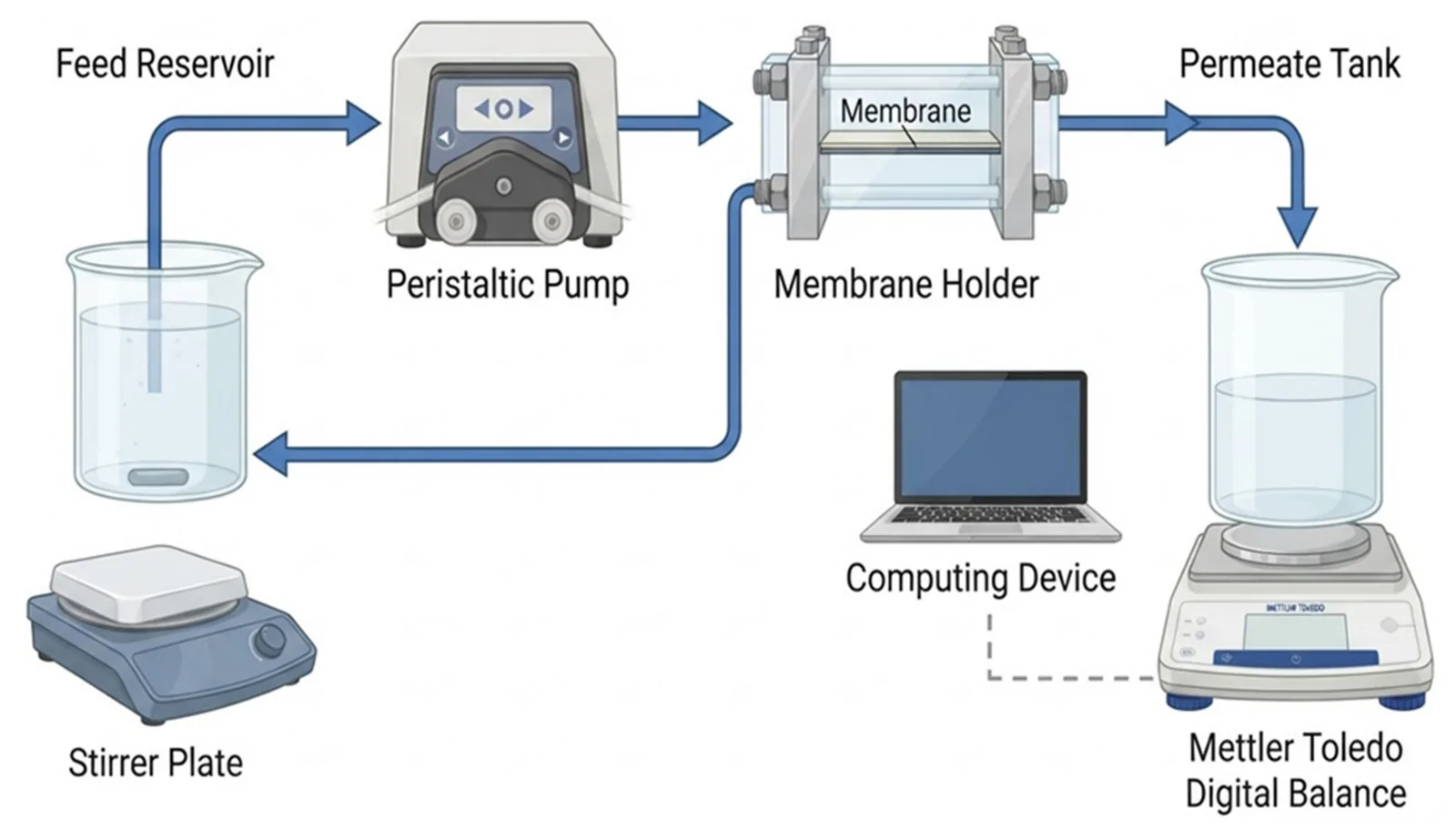
Schematic of the dead-end membrane-filtration rig: feed reservoir on a stirrer plate, peristaltic pump, membrane holder, and permeate tank on a digital balance logged to a computer.

The pure water flux of the 3D-printed membranes was determined in dead-end filtration mode using DI water as the feed at a pump speed of 80 rpm for 30 min. The MP removal experiments were conducted using a laboratory-scale filtration setup (EPDM version, Sterlitech, Auburn, WA, USA) at ambient temperature in a dead-end mode. The membranes were placed in a ceramic membrane disc holder with an active filtration area of 12.57 cm^2^, and the pump was operated at 6 rpm. The permeate volume was automatically recorded using a digital balance (Mettler Toledo, Greifensee, Switzerland) at 1-min intervals. The permeate flux, J (L m^−2^ h^−1^), was calculated from the recorded permeate volume, filtration area, and filtration time. A 1 L feed solution was placed on a magnetic stirrer for continuous mixing, while the permeate vessel was positioned on a digital balance (OHAUS, Parsippany, NJ, USA) to record changes in permeate mass over time. The feed was delivered using a Masterflex L/S peristaltic drive fitted with a Masterflex Easy-Load II pump head (Model 77200-62; Cole-Parmer, Vernon Hills, IL, USA) and L/S 24 tubing (6.4 mm inner diameter), operated at 6 rpm. The experimental filtration setup is shown schematically in Figure 2.

The PSD and mass of particles in the feed and permeate were measured by laser diffraction using a Mastersizer 3000 operated in wet dispersion mode (Malvern Panalytical, Malvern, UK; measurement range 0.01–3500 µm) and, for the sub-micrometre range, by dynamic light scattering using a Zetasizer Nano ZS (Malvern Panalytical, Malvern, UK; measurement range: 0.03 nm–1 µm). The Zetasizer is limited to particles smaller than approximately 1 µm; therefore, the Mastersizer 3000 was used for all experiments following the initial 1500 °C tests. The Mastersizer 3000 software reported particle concentration as volume percentage (vol%). The total volume of MP particles in each analyzed sample was calculated from the reported volume fraction and the known sample volume. The corresponding particle mass was then calculated from the particle volume using the density of the respective polymer (PE or PET). Finally, the MP mass concentration (mg/L) was obtained by dividing the calculated particle mass by the volume of the analyzed sample and applying the appropriate unit conversion. The same procedure was applied to the feed and permeate samples, and the resulting concentrations were used to calculate MP removal efficiency. The following refractive indices were selected from the software library: water = 1.33, 70% ethanol–DI solution = 1.334, PE = 1.52, and PET = 1.63.

Removal efficiency was calculated using the feed and permeate particle concentrations (mg/L), determined from the measured PSD and particle mass, according to Equation (1):

(1) $$R\left(\%\right)=\left(\frac{C_{f}-C_{p}}{C_{f}}\right)\times 100$$

where *C_f_* represents feed concentration (mg/L), *C_p_* is permeate concentration (mg/L), and *R* is removal efficiency (%).

To ensure experimental reliability, all characterization and filtration tests were conducted in triplicate. The results are reported as the mean values of three independent experiments, with standard deviation provided where applicable. Each feed and permeate sample from the individual experiments was analyzed once using the Mastersizer 3000.

## 3. Results

### 3.1. Characterization of 3D-Printed Membranes

**Figure 3:**
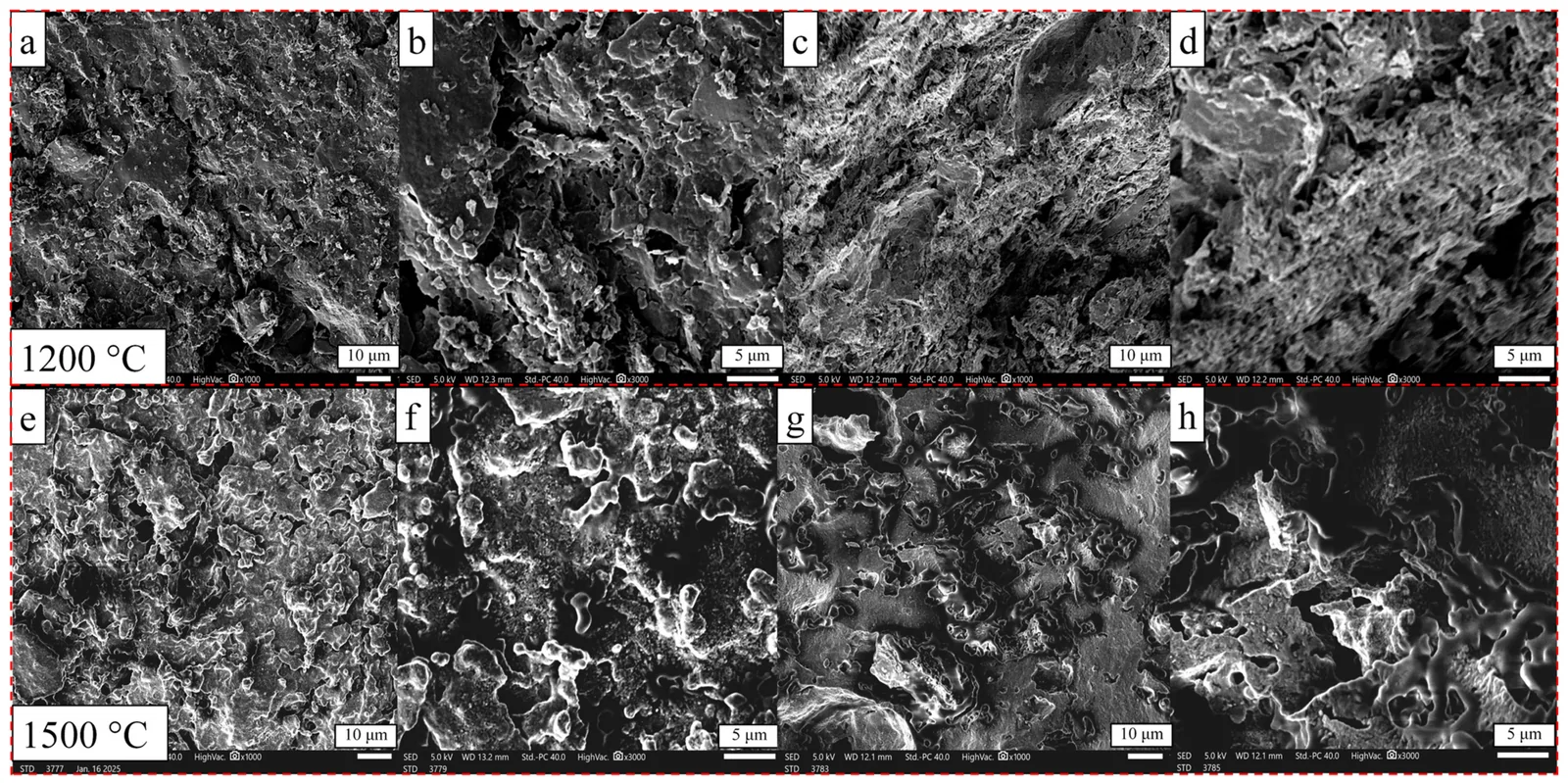
SEM images of the pristine 3D-printed ceramic membrane sintered at 1200 °C (**a**–**d**) and 1500 °C (**e**–**h**): surface morphology (**a**,**b**,**e**,**f**) and cross-sectional morphology (**c**,**d**,**g**,**h**).

The contact angles of the membranes sintered at 1200 °C and 1500 °C were 11.3 ± 0.5° and 52.4 ± 2.3°, respectively, indicating that both membranes were hydrophilic. The higher contact angle of the 1500 °C membrane was attributed to high-temperature densification and vitrification, which reduced the open porosity from 44.8 ± 1.9% to 21.0 ± 0.4% and produced a smoother, less porous surface with fewer accessible surface hydroxyl groups. Both effects reduced capillary uptake of water (consistent with the Wenzel wetting regime, in which surface roughness and porosity amplify the intrinsic hydrophilicity of the aluminosilicate). Consequently, the more vitrified 1500 °C membrane demonstrated lower hydrophilicity. The pure water flux values were high: 4561 ± 16 and 5234 ± 19 L m^−2^ h^−1^ for the membranes sintered at 1200 °C and 1500 °C, respectively. The surface and cross-section morphologies of the pristine 3D-printed membranes sintered at 1200 °C and 1500 °C were investigated using SEM (Figure 3).

**Figure 4:**
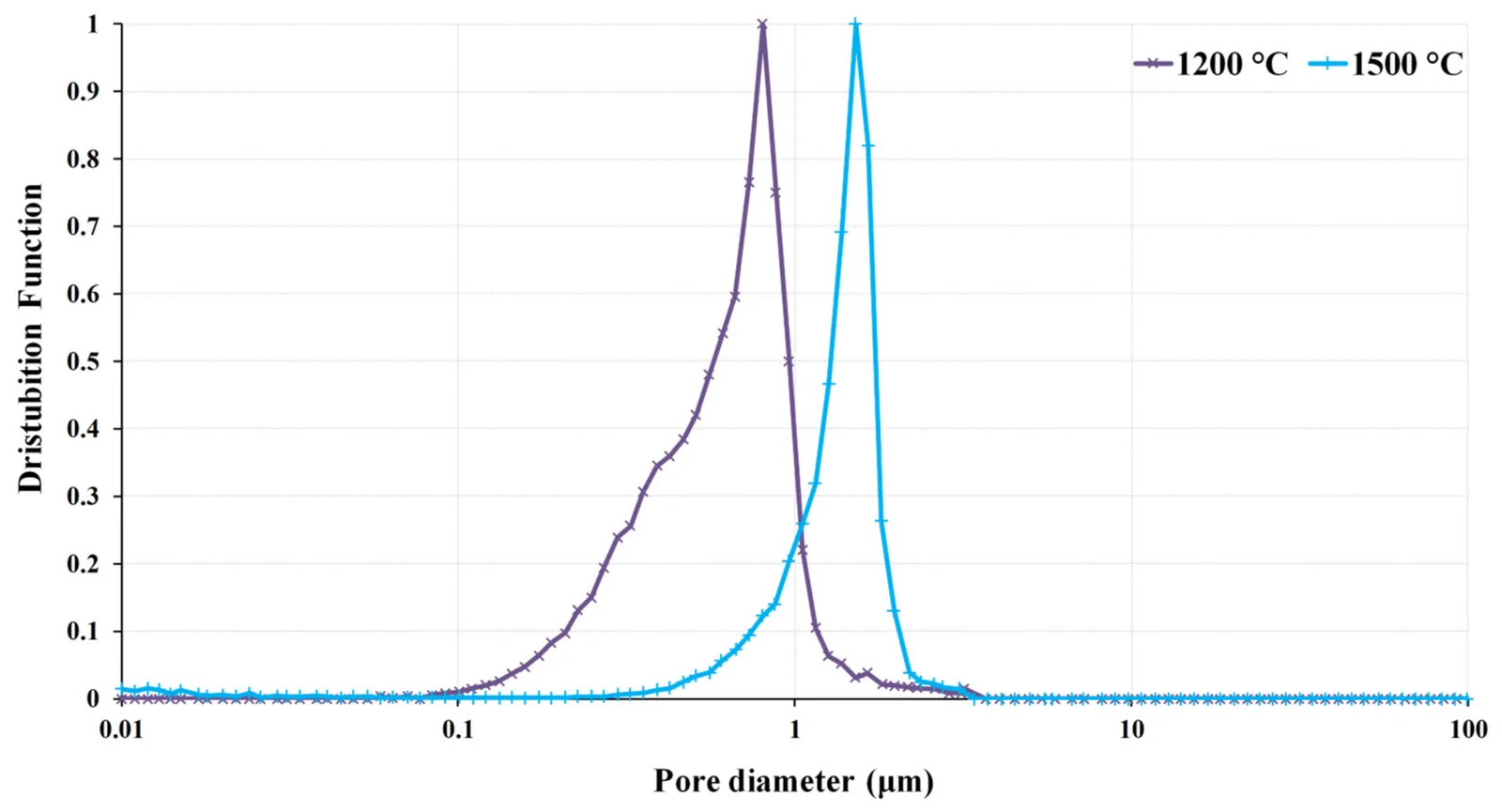
Pore-size distributions of the 3D-printed membranes sintered at 1200 °C and 1500 °C.

Both membranes demonstrated sponge-like structures with asymmetric pores. The open porosity and pore size analyses of 3D-printed membranes showed that the ceramic membranes sintered at 1200 °C had a modal pore size of 0.803 ± 0.005 μm and an open porosity of 44.80 ± 1.9%, whereas the corresponding values for membranes sintered at 1500 °C were 1.517 ± 0.006 μm and 21 ± 1.0%. These results are consistent with previous studies reporting that ceramic membranes sintered at lower temperatures generally exhibit higher porosity [26]. The pore size distributions of the fabricated membranes are shown in Figure 4.

### 3.2. MP Removal by Commercial and 3D-Printed Membranes

First, PE removal experiments were conducted using the 3D-printed membranes to identify the better-performing membrane for subsequent experiments and comparison with the commercial membrane. Both membranes demonstrated complete removal of 10 mg/L PE particles with size ranges of 75–90, 300–355, and 600–710 µm from the 70% ethanol–DI water solution. Only 29.1% of the 1–10 µm PE fraction at an initial concentration of 10 mg/L was removed by the membrane sintered at 1500 °C, whereas the membrane sintered at 1200 °C achieved 60.7% removal. It is important to note that, even in a 70% ethanol–DI water solution, PE particles may still undergo aggregation or flotation during sample preparation and filtration [25]. Such aggregation may affect the measured particle mass concentration by altering the dispersion state of the particles in the analyzed solution. Consequently, the measured removal efficiency may reflect not only membrane retention but also changes in particle aggregation and dispersion during filtration. This effect is particularly relevant to the finest PE fraction, for which particle aggregation may be more pronounced. Therefore, potential changes in particle dispersion should be considered when interpreting the measured removal efficiencies. Moreover, the effect of the ethanol-containing medium on membrane properties and filtration performance was not independently investigated in this study. Thus, potential changes in membrane–liquid interactions, flux, or particle behavior cannot be completely excluded. However, because both the commercial and 3D-printed membranes were evaluated under identical medium composition and operating conditions, the comparison between the two membrane types was conducted under the same experimental conditions. Nevertheless, this limitation should be considered when interpreting the absolute removal and flux values obtained for PE.

**Figure 5:**
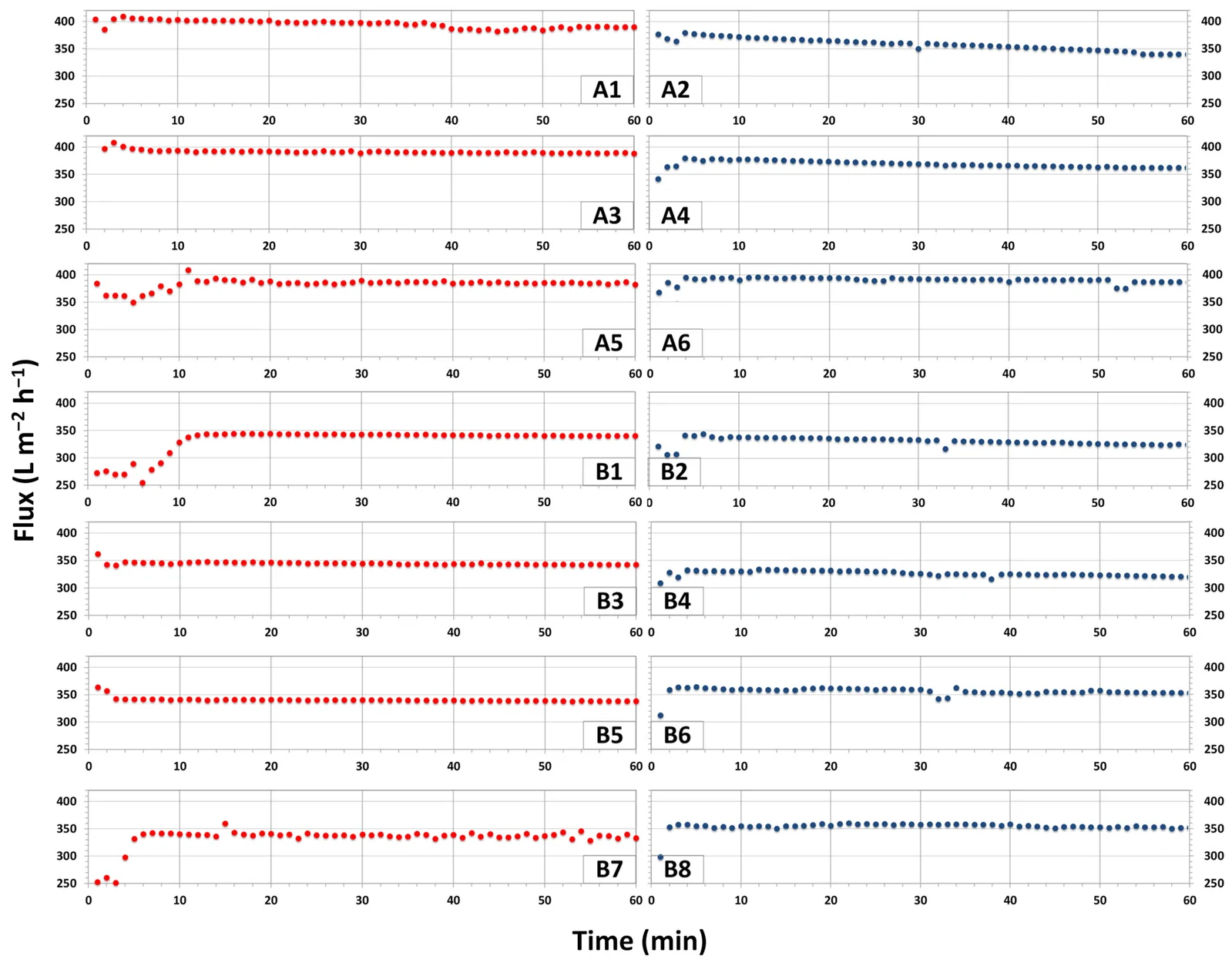
The flux profiles during MP filtration experiments using commercial (red) and 3D-printed (blue ceramic membranes. PET and PE MPs were tested at an initial concentration of 100 mg/L. PET MPs: (**A1**,**A2**) (10–45 µm), (**A3**,**A4**) (45–75 µm), and (**A5**,**A6**) (75–150 µm). PE MPs: (**B1**,**B2**) (1–10 µm), (**B3**,**B4**) (75–90 µm), (**B5**,**B6**) (300–355 µm), and (**B7**,**B8**) (600–710 µm).

These preliminary experiments at 10 mg/L results were used only for membrane selection; all quantitative removal values reported in Table 1 refer to the 100 mg/L experiments. The flux profiles of the commercial and 3D-printed membranes during the MP removal experiments are presented in Figure 5.

The permeate flux profiles for PET (A1–A6) and PE (B1–B8) microplastic filtration experiments showed generally stable membrane performance during the 60 min filtration period. The commercial membrane (red) exhibited an initial adjustment period, followed by flux stabilization, particularly during the PE experiments. In comparison, the 3D-printed ceramic membrane (blue) exhibited relatively stable fluxes across most experiments, with only minor fluctuations and occasional short-term decreases. Overall, both membranes maintained relatively consistent filtration performance, although the flux magnitude and stabilization behavior varied depending on the microplastic type and particle size. Table 1 summarizes the MP removal efficiencies of the ceramic membranes.

The MP removal performance of the commercial (0.45 μm) and 3D-printed (0.80 μm) ceramic membranes was evaluated using PE and PET particles at an initial concentration of 100 mg/L. The commercial membrane achieved complete (100 ± 0.0%) removal across all tested PE and PET size fractions. In contrast, the 3D-printed membrane exhibited pronounced size-dependent removal, with removal efficiency increasing with particle size. For PE, removal increased from 58.3 ± 3.6% for the smallest particles (1–10 μm) to 100% for the 75–90, 300–355, and 600–710 μm fractions. For PET, removal was 80.8 ± 0.9% for the 10–45 μm fraction, 94.9 ± 1.8 for the 45–75 μm fraction, and 100% for the 75–150 μm fraction.

It should be noted that the 0.80 μm pore diameter of the 3D-printed membrane represents the modal pore diameter obtained from the peak of the MIP pore-size distribution and should not be considered the effective filtration pore size or a direct pore-size cutoff. The actual transport and retention of MPs within and through the membrane may be influenced by the overall pore-size distribution, pore connectivity, tortuosity, particle interception, depth filtration, surface retention, cake formation, and particle–membrane interactions. Therefore, MP removal cannot be attributed solely to direct size exclusion based on the modal pore diameter. In particular, the 58.3 ± 3.6 removal observed for the 1–10 μm PE fraction should not be interpreted as a direct consequence of the relationship between the nominal particle-size range and the 0.80 μm modal pore diameter.

Nevertheless, the increase in PE removal from 58.3 ± 3.6 for the 1–10 μm fraction to 100% for particles ≥75 μm indicates a strong particle-size dependence of membrane retention. The complete retention of the larger particles is consistent with their increased probability of being intercepted and retained within the porous membrane structure. For the finest PE fraction, particle transport through the interconnected pore network may have been more likely, while particle interception, depth filtration, and surface deposition may have contributed to the observed partial removal. It is also important to note that, even in a 70% ethanol–DI water solution, PE particles may still undergo aggregation or agglomeration during sample preparation and filtration, which may influence their dispersion and retention behavior.

The higher removal observed for PET compared with the finest PE fraction may be associated with differences in particle-size distribution, density, morphology, and surface characteristics, as well as the broader PET size range tested. In particular, the irregular, non-spherical morphology of the PET fragments, in contrast to the smooth spherical morphology of the PE microspheres, may promote particle interception and retention at the membrane surface, although this contribution cannot be fully separated from the simultaneous differences in particle size. Overall, these results demonstrate that the 3D-printed ceramic membrane can effectively retain relatively large MPs, although its removal efficiency is more sensitive to particle size than is that of the commercial membrane.

### 3.3. Structural and Chemical Characterization of Membranes Before and After MP Filtration

**Figure 6:**
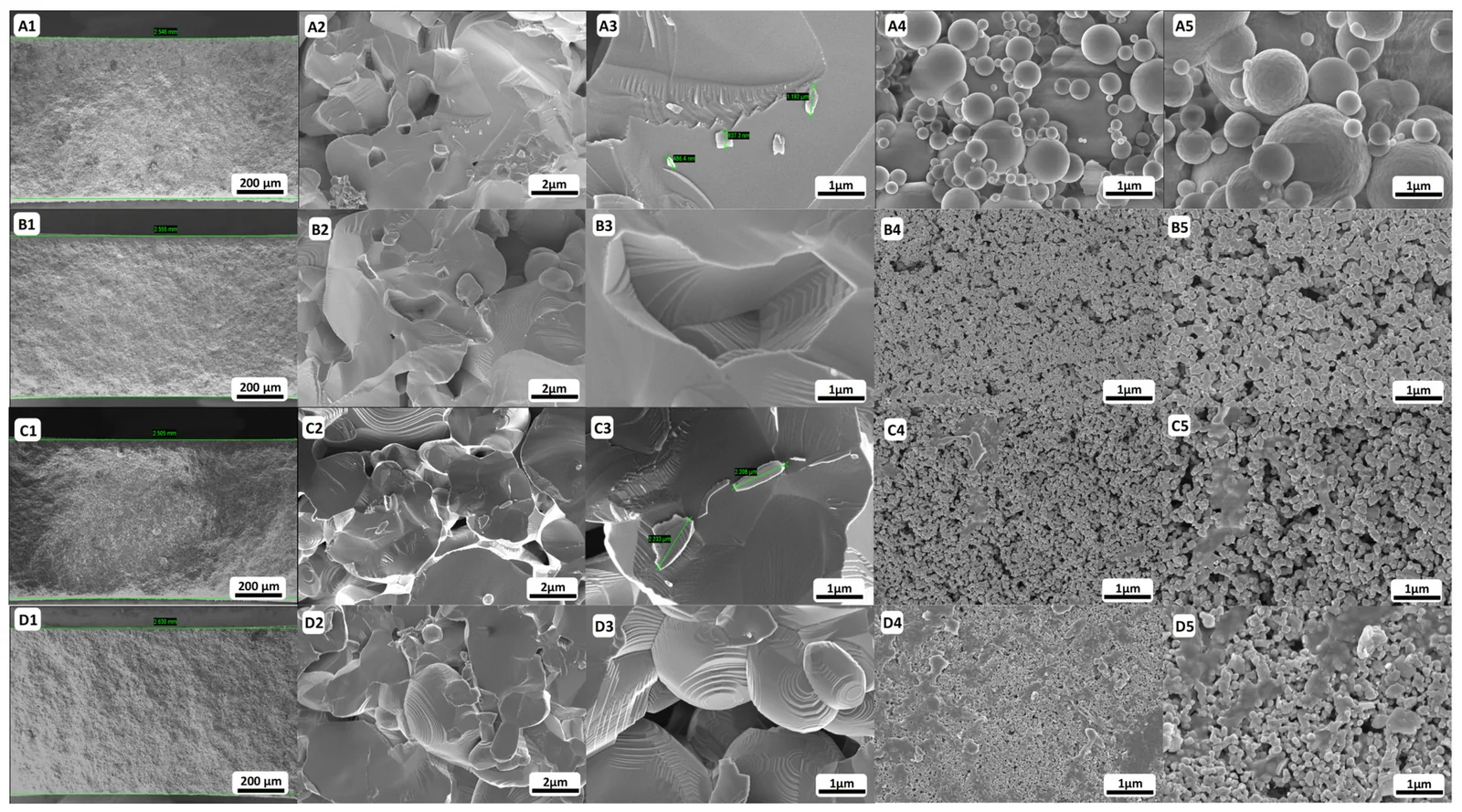
SEM images of Tami-based samples containing PE and PET MPs: (**A1**–**A5**) PE MPs (1–10 µm); (**B1**–**B5**) PE MPs (600–710 µm); (**C1**–**C5**) PET MPs (10–45 µm); and (**D1**–**D5**) PET MPs (75–150 µm). Cross-sectional and surface morphologies are shown at different magnifications (from left to right).

**Figure 7:**
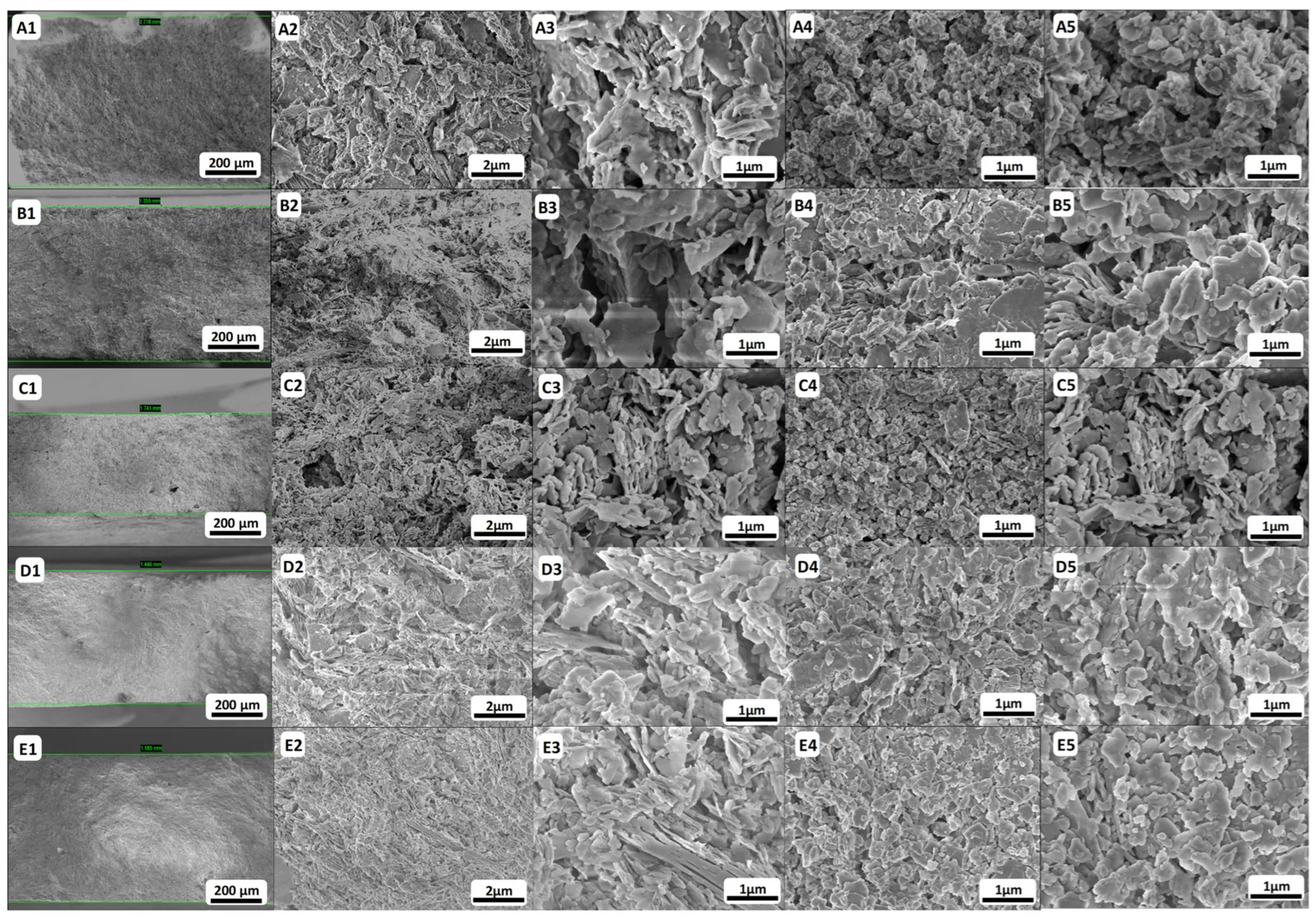
SEM images of 3D-printed samples containing PE and PET MPs of different particle-size ranges: (**A1**–**A5**) pristine 3D; (**B1**–**B5**) PE MPs (1–10 µm); (**C1**–**C5**) PE MPs (600–710 µm); (**D1**–**D5**) PET MPs (10–45 µm); and (**E1**–**E5**) PET MPs (75–150 µm). Cross-sectional and surface morphologies are shown at different magnifications (from left to right).

SEM–EDS analyses of both membranes are presented in Figure 6 and Figure 7; and the complete set of EDS spectra is provided in the Supplementary Material (Figure S1).

The TAMI membrane had a wall thickness of ~2.55 mm and a dense, well-sintered microstructure consisting of large faceted grains overlaid by a fine granular selective layer composed of 0.2–2 µm rounded particles. EDS analysis of this layer showed 48.6–54.0 wt% Ti and 39.9–43.5 wt% O (O/Ti = 2.2–2.7), consistent with TiO_2_ composition; no zirconium was detected. The 3D-printed kaolin–rice-husk (4:1) membrane showed a homogeneous, monolithic cross-section and an irregular, platelet-like surface composed of interlocking lamellae with open, interconnected voids, reflecting the layered kaolin precursor and the porosity generated by rice-husk burnout. Its composition was predominantly aluminosilicate (50.1–55.1 wt% O, 18.7–24.2 wt% Si, and 17.4–20.8 wt% Al; Si/Al = 1.03–1.13). The slight increase in the Si/Al ratio relative to kaolinite stoichiometry (1.0) may reflect the contribution of silica from the rice husk, with minor Fe, K, and Ca (≤0.5 wt%) present as natural impurities. Bright flake-like features of approximately 2.23 µm were observed on the membrane surface (Figure 7, C3) and were attributed to retained polymer fragments. In particle-free regions, carbon remained uniformly low (4.5–8.3 wt%, including 7.2 wt% in the pristine membrane), consistent with adventitious surface carbon. In contrast, a particle-bearing region of the TAMI membrane exposed to 10–45 µm PET showed 23.9 wt% C, approximately four times the background level, accompanied by a decrease in Ti to 39.2 wt% and an increase in the O/Ti ratio to 2.74. These changes are consistent with attenuation of the TiO_2_ signal by an overlying oxygen-containing organic layer and provide additional evidence of polymer retention on the membrane surface. Retained particles were confined to the outer surface, with no penetration into the support observed at the magnifications examined. For the coarse fractions, these observations are consistent with retention predominantly through surface size exclusion; for the fine PE fraction (1–10 µm), the incomplete removal reported in Section 3.2 indicates that surface capture was only partial and that other retention and transport mechanisms may have contributed.

**Figure 8:**
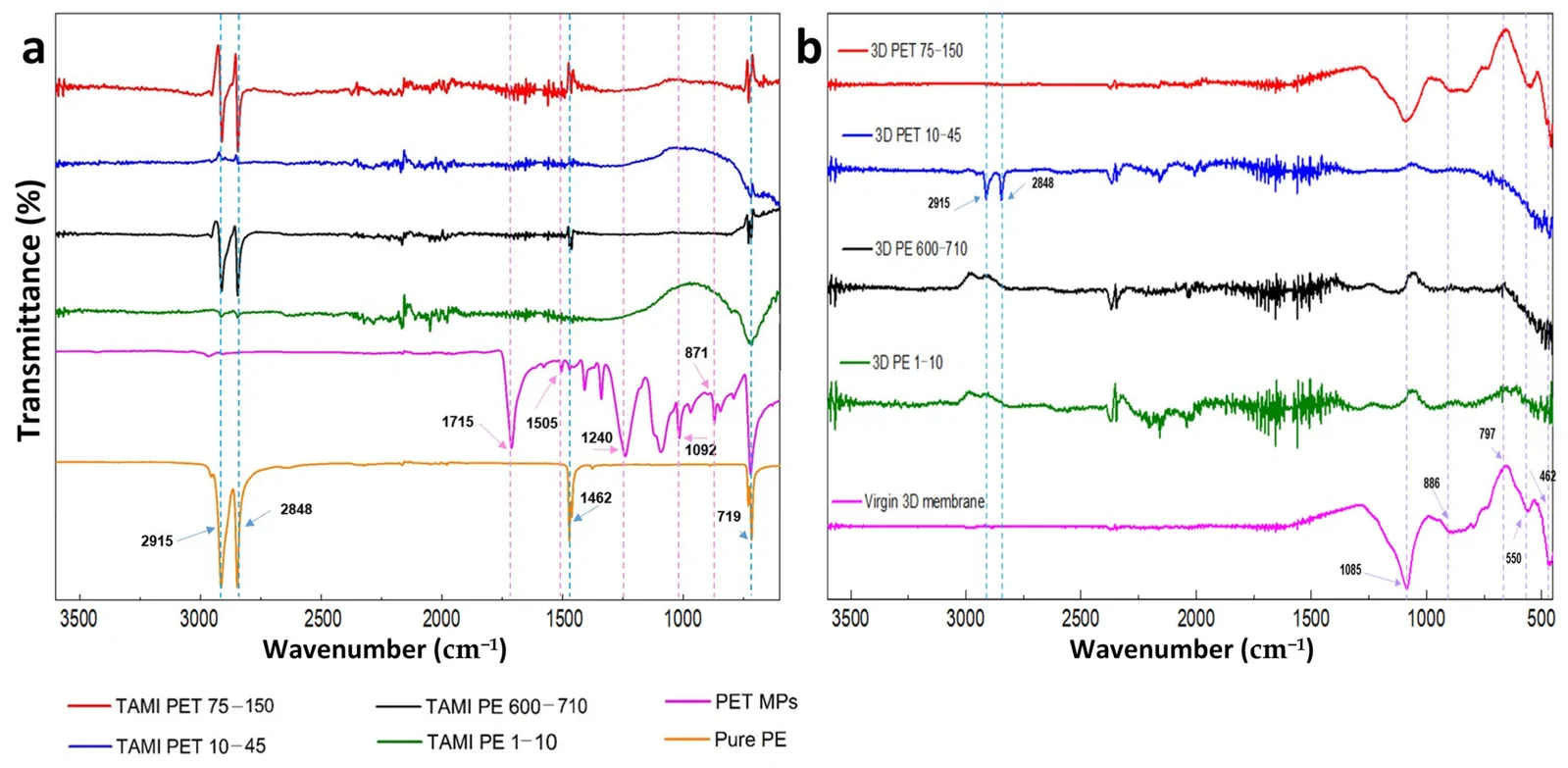
ATR-FTIR spectra of (**a**) PE and PET particles retained on the TAMI commercial ceramic membrane, with pure PE and PET references; and (**b**) the 3D-printed rice husk–kaolin (1:4) membrane before and after filtration. Dashed lines mark the diagnostic polymer and silicate bands. Spectra are baseline-corrected and vertically offset for clarity.

ATR-FTIR spectroscopy was employed to identify retained MP particles and to characterize the composition of the fabricated membrane (Figure 8).

In panel (a), particles recovered from the TAMI commercial ceramic membrane displayed the characteristic absorption bands of polyethylene at 2915 and 2848 cm^−1^, assigned to CH_2_ asymmetric and symmetric stretching, respectively, together with CH_2_ bending at 1462 cm^−1^ and CH_2_ rocking at 719 cm^−1^, in agreement with the pure PE reference. A weak but identifiable PET signature was observed in the 75–150 µm fraction, comprising the ester carbonyl stretching band at 1715 cm^−1^, the C(=O)–O stretching mode at 1240 cm^−1^ and the aromatic C–H out-of-plane deformation band at 721 cm^−1^, consistent with the corresponding bands of the PET reference at 1715, 1505, 1240, 1092 and 871 cm^−1^. The reduced intensity of the PET bands relative to the reference may be attributed to limited particle–crystal contact and the small effective sampling area of the ATR element, which may have limited spectral confirmation for the finer size fractions. Panel (b) presents the spectra recorded for the 3D-printed kaolin–rice-husk (4:1) membrane. The pristine membrane exhibited the silicate framework characteristic of the calcined aluminosilicate body, with an intense Si–O–Si asymmetric stretching band at 1085 cm^−1^, the corresponding Si–O–Si bending mode at 462 cm^−1^, Si–O symmetric stretching at 797 cm^−1^ and a weaker band at 886 cm^−1^ attributed to residual Si–OH/Al–OH surface groups. Following filtration, the membrane retained this ceramic fingerprint while additionally exhibiting the CH_2_ stretching bands at 2912 and 2845 cm^−1^, which were absent in the pristine membrane. Since ATR spectroscopy predominantly probes the near-surface region of the sample, the detection of these polymer bands supports the presence of captured particles at or near the membrane surface. For the coarse fractions confirmed spectroscopically (PE 600–710 µm and PET 75–150 µm), whose dimensions greatly exceed the modal pore diameter of the 3D-printed membrane, this observation is consistent with retention by surface size exclusion. This interpretation should not be directly extended to the fine PE fraction (1–10 µm), for which removal was incomplete (58.3 ± 3.6; Section 3.2). As discussed above, the modal pore diameter obtained by MIP does not represent a direct filtration cutoff; therefore, retention of this fine fraction may involve a combination of particle transport through the interconnected pore network and retention mechanisms such as interception, depth filtration, surface deposition, and particle–membrane interactions rather than size exclusion alone.

**Figure 9:**
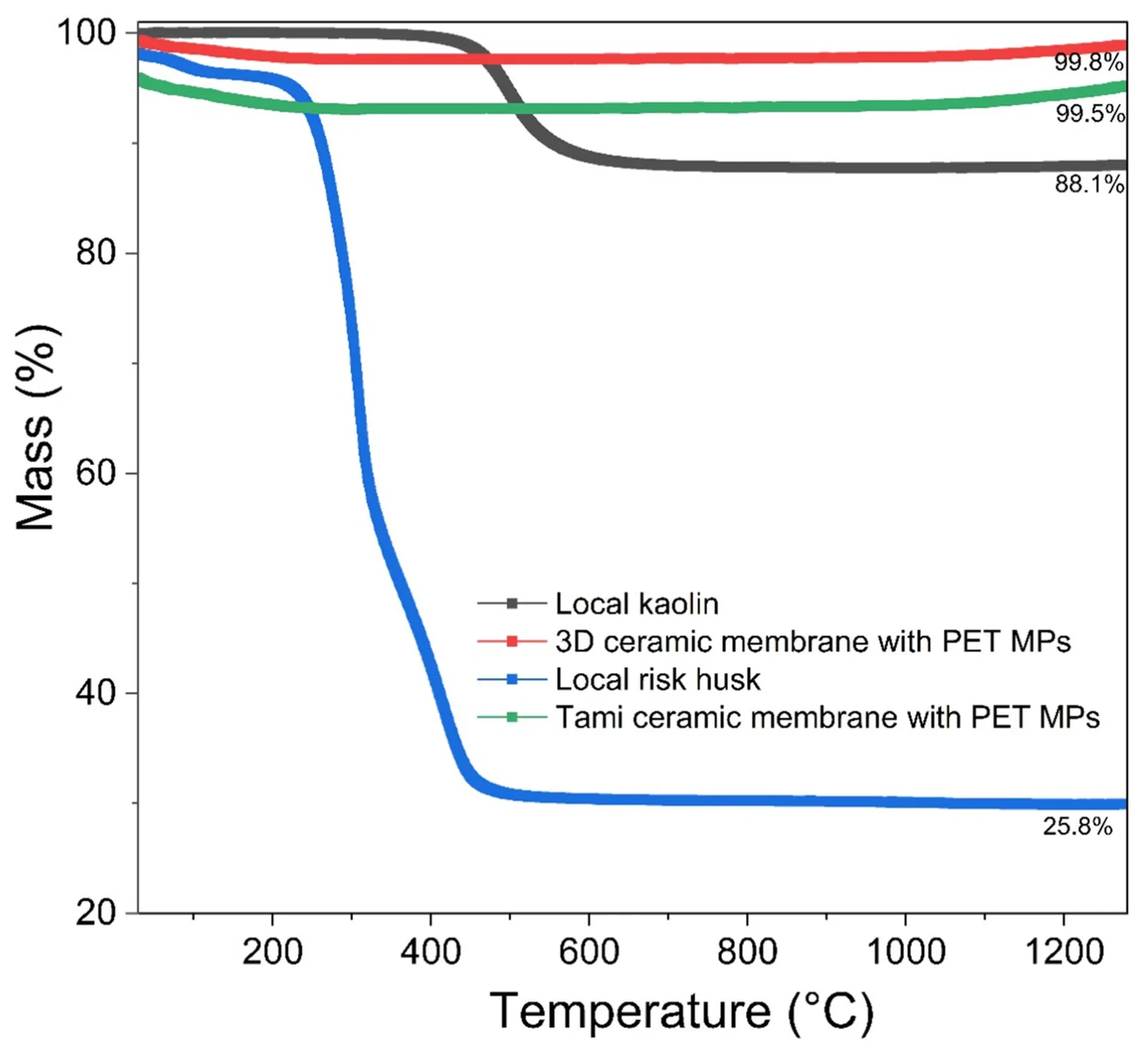
TGA curves illustrating the thermal decomposition behavior of local kaolin, local rice husk, 3D-printed ceramic membrane and commercial Tami membrane containing PET microplastics.

TGA of the raw precursors and sintered membranes is shown in Figure 9.

Local kaolin was stable up to approximately 450 °C and then lost 11.9% of its mass between 450 and 650 °C, corresponding to the dehydroxylation of kaolinite to form metakaolin. This value approaches the theoretical mass loss of 13.96% for pure kaolinite, indicating a predominantly kaolinitic clay with a minor non-clay fraction. Local rice husk showed a small initial mass loss below 200 °C attributed to adsorbed moisture, followed by major decomposition between ~250 and 450 °C associated with the thermal degradation of hemicellulose, cellulose, and lignin, leaving a residue of 25.8%, which was attributed to silica-rich ash and served as a silica source in the membrane formulation. In contrast, the PET-loaded 3D-printed and commercial TAMI membranes retained 99.8% and 99.5% of their mass, respectively, over the full temperature range, consistent with the effective removal of the organic components, including the rice-husk pore-forming material, during sintering and with the high thermal stability of the fired ceramic bodies. No discrete mass-loss step was observed in the PET degradation range (400–450 °C), indicating that the retained microplastic fraction was insufficient to produce a detectable TGA signal under the applied experimental conditions.

## 4. Discussion

The dominant factor controlling membrane performance was the sintering temperature, primarily through its effects on porosity and pore-size distribution. Increasing the sintering temperature of kaolin-based ceramics promotes densification, as liquid-phase and solid-state sintering induce grain growth and shrinkage, thereby decreasing open porosity and reducing both the number and connectivity of pores [27,28]. For raw kaolin, open porosity has been reported to decrease from 34–38% at 1000 °C to below 3% at 1500 °C as vitrification and mullite formation proceed [27]. The present results are consistent with this general trend.

Firing at 1500 °C promoted grain growth and vitrification, resulting in a coarser pore structure and a decrease in open porosity to 21%. The shift toward larger pores may have contributed to the lower retention of fine MPs, whereas the substantial reduction in open porosity and pore connectivity may have restricted permeate transport and promoted rapid surface fouling, resulting in poor fine-MP removal and less stable filtration performance. In contrast, firing at 1200 °C preserved a finer, highly porous, and interconnected structure (modal pore diameter: 0.80 µm; open porosity: 44.8%), comparable to the 30–55% porosity reported for other kaolin–rice-husk ceramics sintered near 1200 °C [19,29,30]. This more open pore structure provided favorable conditions for stable permeate transport while maintaining effective retention of the tested MP fractions.

The higher removal of larger MPs by the 3D-printed membrane indicates that retention was strongly influenced by particle size, although the MIP-derived modal pore diameter should not be interpreted as a direct filtration cutoff. The observed removal likely resulted from a combination of size exclusion, particle interception, depth filtration, surface retention, and cake formation rather than size-based sieving alone.

The comparison with the commercial TAMI membrane should be considered a benchmark rather than a direct like-for-like comparison, as the two membranes differ in material composition, morphology, thickness, pore structure, and membrane architecture. In particular, the TAMI membrane incorporates a fine selective surface layer, whereas the 3D-printed membrane has a more porous monolithic structure. Therefore, the observed differences in MP retention and permeate flux cannot be attributed solely to differences in pore size.

### 4.1. Cost Estimation

To establish whether the 3D-printed rice-husk–kaolin membrane offers a genuine economic advantage over commercially available alternatives, the costs associated with its fabrication were estimated and compared with the purchase cost of a commercial ceramic disc used for the same filtration function. The assessment distinguishes costs directly associated with fabrication, including raw materials, labor, energy, and maintenance, from those associated with the capital equipment used, in line with established laboratory-scale techno-economic assessment practices [31,32]. The cost estimates are based on a single fabrication batch of 20 discs, each 40 mm in diameter and having an effective filtration area of 12.57 × 10^−4^ m^2^, and are expressed in United States dollars using an exchange rate of 447 KZT per USD.

Kaolin was obtained locally at USD 0.50 kg^−1^ and rice husk was purchased at USD 4.50 kg^−1^. Preparation of a 300 g of dry powder at a rice husk-to-kaolin mass ratio of 1:4 therefore required USD 0.39 in precursor materials per batch. The apparently higher unit price of rice husk primarily reflects its very low bulk density when purchased in small retail quantities. As rice husk is an abundant agricultural residue with low intrinsic value, its unit cost may be substantially reduced when sourced in larger quantities; however, this represents a scale-up projection rather than an experimentally demonstrated cost reduction.

Operator labor was valued based on the doctoral stipend prevailing at the host institution, USD 500 month^−1^, equivalent to USD 2.84 h^−1^ based on a 22-day working month and an 8 h working day. A single batch required approximately one full day of supervised activity, encompassing rice-husk washing, mill loading, paste preparation, printing, and furnace loading. Milling, ambient drying of the green bodies, and the sintering cycle itself proceeded without operator attendance and were accordingly excluded from the labor-cost calculation. Consumables and ancillary laboratory overheads were estimated at 20% of the operator labor cost, following the proportion adopted for the economic assessment of laboratory-scale processes [33].

The electrical energy drawn by each item of equipment was evaluated as Equation (2) [31]:

(2) $$C_{e}=Pn_{h}t$$

where *P* is the rated power of the unit, *n_h_* the number of hours it operates during a run, *t* the prevailing price of electricity, taken as USD 0.0629 kWh^−1^, and *C_e_* the resulting energy cost.

Rated powers were taken from the respective manufacturers’ technical documentation [4,5,6]. A complete run consumes 102.4 kWh, of which the 24 h sintering dwell alone accounts for 84%. Full particulars are given in Table S1 of the Supplementary Material.

Depreciation of the fabrication equipment was treated on a straight-line basis according to Equation (3) [32]:

(3) $$V_{d}=\frac{{(V}_{o}-V_{s})a}{n_{s}}$$

where *V_0_* denotes the purchase price, *V_s_* the residual value at the end of the asset’s useful life, assumed to be 10% of the original outlay, *n_s_* the useful life, assumed to be 10 years, *a* the elapsed years of service, and *V_d_* the depreciation accrued over that interval [32].

Acquisition costs were reconstructed from current supplier quotations [34,35,36] and adjusted for inflation to the year of purchase. None of these instruments was used exclusively for membrane fabrication; therefore, equipment depreciation, together with an annual maintenance allowance of 2% of the asset value, was apportioned according to the operating time required for each fabrication batch, assuming 2000 available operating hours per year. The resulting cost allocations are presented in Table S2.

Adding these contributions yields USD 59.64 for a run of 20 discs, corresponding to estimated unit fabrication cost of USD 2.98, or USD 2373 per square metre of filtration area. The commercial TAMI ceramic disc against which the fabricated membrane was benchmarked in this work (0.45 µm nominal pore size, 47 mm diameter) is supplied at 54.45 USD per unit, equivalent to 31,383 USD m^−2^ [37]. Accordingly, the estimated fabrication cost of the 3D-printed membrane was approximately 18.3 times lower on a per-disc basis and 13.2 times lower per unit filtration area than the purchase cost of the commercial membrane. These results indicate the potential economic advantage of the proposed fabrication approach, particularly with respect to the low cost of the precursor materials [38].

**Figure 10:**
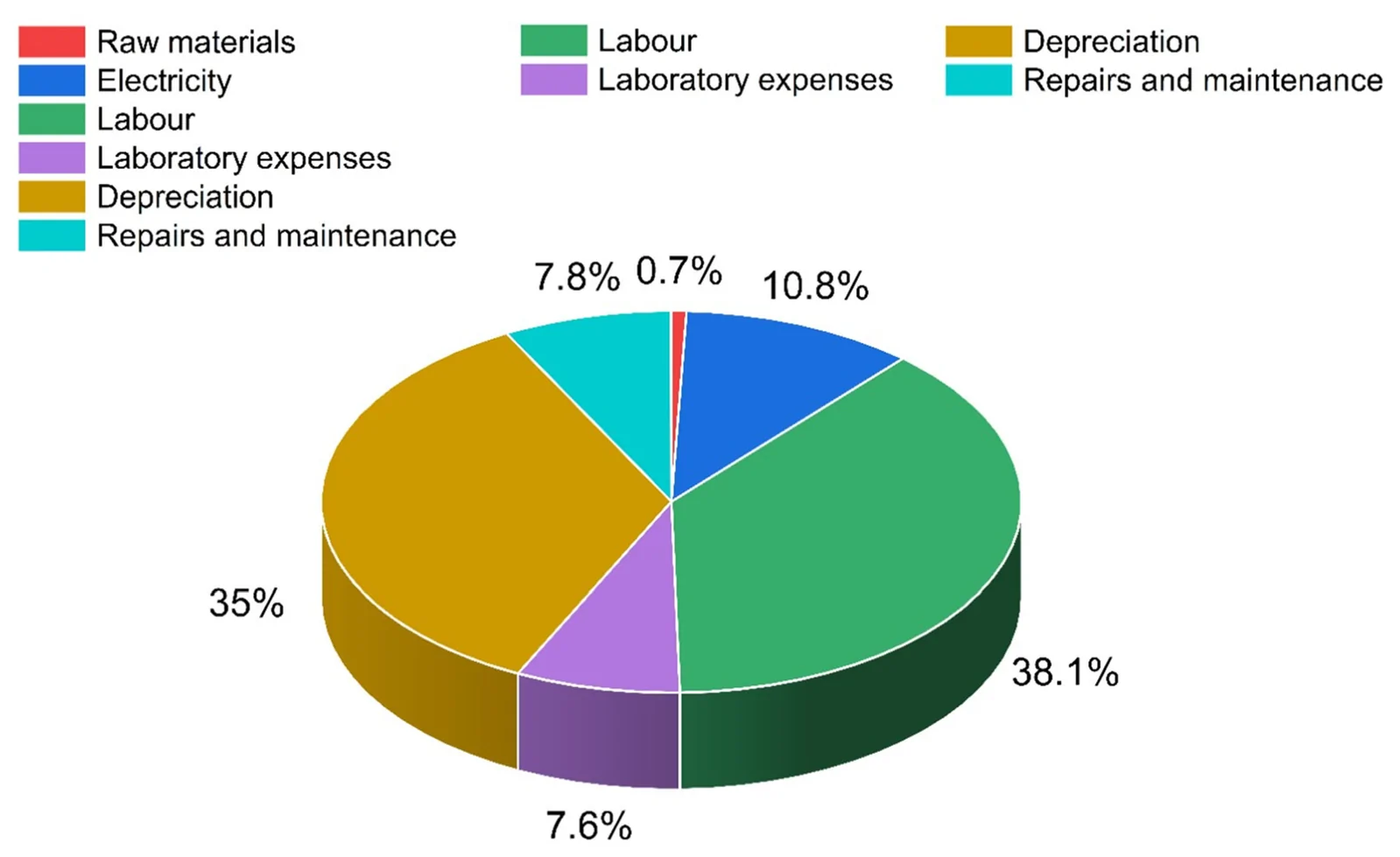
Contribution of each cost head to the total fabrication cost, expressed as a percentage. Values refer to one production run of 20 discs (40 mm diameter, 12.57 × 10^−4^ m^2^ each).

Figure 10 shows the relative contribution of each cost category, with the supporting calculations provided in Table S3. Precursors account for only 0.7% of the total, indicating that raw-material costs make only a minor contribution to the estimated fabrication cost when low-cost kaolin and waste-derived rice husk are used as precursors. Most of the remaining cost is associated with operator labor and capital recovery. With increasing production batch size, the estimated unit cost is expected to decrease as certain fixed or semi-fixed costs, including equipment operation, labor, depreciation, and maintenance, are distributed over a larger number of membranes. This represents a projected cost trend based on the assumptions described in the Supplementary Material and was not experimentally validated through large-scale production. Extrusion-based 3D printing may further support increased production throughput, as previous estimates indicate a five- to twenty-fold increase in productivity compared with conventional dry pressing for 40 mm disc membranes. In addition, unused extruded paste, rejected green parts, and printing residues can potentially be recovered and returned to the mixing stage, thereby reducing material waste.

### 4.2. Practical Implications and Limitations

Two key advantages of the developed manufacturing strategy should be highlighted: (i) its high productivity and process flexibility, and (ii) the potential for recycling the microextruded paste generated during printing, including material from defective components and printing residues. In terms of productivity, for the fabrication of single-layer button membranes with a diameter of 40.0 mm, the estimated production rate was approximately 5–20 times higher, depending on the printing speed, than that estimated for conventional batch fabrication methods such as dry pressing. In addition, preliminary experiments demonstrated that the microextruded paste could be reused after printing by reintroducing the recovered material into the continuous planetary mixing process. These features may be advantageous for future scale-up and improved resource efficiency in membrane manufacturing, as they have the potential to reduce material waste while increasing production throughput. However, these benefits remain to be validated under larger-scale manufacturing conditions.

Within the context of the wider literature, the 1200 °C membrane represents a promising low-cost alternative for specific MP filtration applications rather than a direct replacement for fine-pore commercial ceramic membranes. Ceramic MF used as a polishing step has been reported to remove more than 72% of MPs, while combined or tertiary treatment trains can achieve removal efficiencies of 95–99% [14,39,40]. In the present study, the commercial TAMI membrane achieved 100% removal of the tested PET fractions down to 10 μm and PE fractions down to 1 μm. The 3D-printed membrane achieved complete removal of intermediate and coarse MP fractions, but its performance decreased for the smallest PE fraction, indicating that its pore structure provides less effective retention of the finest tested particles. Therefore, potential near-term applications include its use as an inexpensive, waste-derived pre-filter or as a macroporous support for a finer active layer, taking advantage of its high pure-water flux and low estimated fabrication cost. The use of rice husk simultaneously provides a potential route for valorizing an abundant agricultural residue and contributes to pore formation, while extrusion-based 3D printing enables tooling-free fabrication with considerable geometrical flexibility and the potential for reproducible production [23,41,42]. These attributes may be particularly relevant to resource-constrained and understudied regions such as Central Asia, where MP contamination has been documented but affordable, locally manufacturable treatment options remain limited [43].

Several limitations of the present study should be acknowledged and considered in future work. First, the reported pore sizes of the commercial and 3D-printed membranes represent different pore-size metrics. The 0.45 µm value for the commercial TAMI membrane is the manufacturer-specified nominal pore size, whereas the 0.80 µm value for the 3D-printed membrane represents the modal pore diameter determined by MIP. These values should therefore not be considered directly equivalent, and differences in MP retention between the two membranes cannot be attributed solely to their reported pore sizes. Second, the fine-PE experiments relied on 70% ethanol for dispersion; this medium is not representative of environmentally relevant aqueous conditions and introduced bubble-related measurement artifacts. Therefore, the corresponding removal values should be regarded as semi-quantitative. Third, feed and permeate concentrations were calculated from the particle mass reported by the Mastersizer software and the volume of the analyzed sample, rather than being independently verified by direct particle counting or an alternative mass-based analytical method. Although the filtration experiments were conducted in triplicate, each feed and permeate sample from the individual experiments was analyzed once by Mastersizer; therefore, the analytical uncertainty associated with the concentration measurements could not be independently quantified. Fourth, the mass of captured MP was below the detection limit of TGA (Figure 9), so polymer capture was confirmed qualitatively by SEM–EDS and ATR-FTIR but not quantified gravimetrically.

Future work should employ mass-balance approaches or techniques such as pyrolysis–GC/MS to enable absolute quantification. Fifth, the filtration experiments were conducted using single-polymer suspensions under controlled laboratory conditions and in dead-end mode; real water matrices may contain mixed polymers, natural organic matter, and colloids that can influence membrane fouling and MP retention. The results demonstrate the potential of the low-cost 3D-printed kaolin–rice-husk membrane for the retention of relatively large microplastic particles under the laboratory conditions investigated. Although its high porosity and low estimated fabrication cost may make it a promising candidate for use as a pre-filter or macroporous support material, these applications remain prospective and require further validation using real water matrices, mixed-polymer suspensions, continuous-flow systems, and larger-scale operation. Future work should therefore focus on optimizing the membrane pore structure through approaches such as finer rice-husk milling, the use of pre-calcined kaolin, or multilayer/asymmetric membrane designs. Further studies should also include comprehensive membrane characterization; replicated analytical measurements with quantified uncertainty; mass- or number-based MP quantification using environmentally relevant dispersion conditions; and evaluation of cross-flow operation, long-term fouling, cleaning and regeneration cycles, performance in real water matrices, and larger-scale production.

## 5. Conclusions

Low-cost porous ceramic membranes were successfully fabricated by extrusion-based 3D printing of a kaolin–rice-husk paste and evaluated for the removal of PE and PET microplastics from water. Sintering temperature strongly influenced the membrane structure and performance, with the membrane sintered at 1200 °C exhibiting higher porosity and a smaller pore size than that sintered at 1500 °C. Both membranes remained hydrophilic and exhibited high pure-water fluxes. The 1200 °C membrane demonstrated effective size-dependent MP retention, achieving 58.3 ± 3.6% removal of PE particles in the 1–10 μm range and 100% removal for the 75–90, 300–355, and 600–710 μm PE fractions. For PET, removal efficiencies of 80.8 ± 0.9%, 94.9 ± 1.8%, and 100% were achieved for the 10–45, 45–75, and 75–150 μm fractions, respectively. In comparison, the commercial 0.45 μm TAMI ceramic membrane achieved 100% removal across all tested PE and PET fractions. Importantly, the 3D-printed membrane had an estimated fabrication cost of only USD 2.98 per membrane, compared with a purchase cost of USD 54.45 per membrane for the commercial TAMI ceramic membrane. Although these values represent different cost bases, the comparison highlights the potential economic advantage of the proposed fabrication approach. Overall, the results indicate that the 3D-printed membrane can effectively retain intermediate and coarse MPs while providing high pure-water flux and a low estimated fabrication cost. The combination of high porosity, hydrophilicity, MP retention, and the use of low-cost precursors supports its potential application as a pre-filter or macroporous support for a finer selective layer. However, these applications remain prospective and require validation under environmentally relevant and larger-scale operating conditions. Further optimization of paste composition, printing architecture, and pore-size distribution is required to improve the retention of sub-10 μm MPs while maintaining favorable membrane permeability. Future studies should additionally evaluate real water matrices, mixed-polymer suspensions, continuous-flow operation, long-term fouling, and cleaning and regeneration performance. Such investigations will help establish the practical feasibility of locally manufacturable 3D-printed ceramic membranes for MP control, including their potential application in regions such as Central Asia.

## Figures and Tables

**Table 1:** Removal efficiencies of PE and PET microplastics by the commercial and 3D-printed ceramic membranes at an initial feed concentration of 100 mg/L.

Polymer	Particle Size (µm)	Commercial Membrane (0.45 μm), Removal (%)	3D-Printed Membrane (0.80 μm), Removal (%)
PET	10–45	100 ± 0.0	80.8 ± 0.9
	45–75	100 ± 0.0	94.9 ± 1.8
	75–150	100 ± 0.0	100 ± 0.0
PE	1–10	100 ± 0.0	58.3 ± 3.6
	75–90	100 ± 0.0	100 ± 0.0
	300–355	100 ± 0.0	100 ± 0.0
	600–710	100 ± 0.0	100 ± 0.0

## Data Availability

The raw data supporting the conclusions of this article will be made available by the corresponding author upon request.

## Supplementary Materials

The following supporting information can be downloaded at https://www.mdpi.com/article/10.3390/membranes16100346/s1: Figure S1: EDS map sum spectra and elemental compositions (wt%) of the TAMI commercial membrane and the 3D-printed rice husk–kaolin membrane, before and after filtration of PE and PET microplastics of different size fractions.; Table S1: Electrical energy consumed per fabrication run of 20 membranes; Table S2: Equipment acquisition values, annual depreciation and allocation per fabrication run; Table S3: Breakdown of the total fabrication cost; Table S4: Comparison with the commercial ceramic membrane used as a benchmark; Table S5: Fabrication process parameters; Table S6: Elemental composition of the kaolin precursor determined by ICP-OES after acid digestion (HNO_3_ 5 mL, H_2_SO_4_ 1 mL, HClO_4_ 1 mL, HF 2 mL; neutralized with 5 mL boric acid), reported as blank-corrected means (n = 3). Solid-phase content was obtained from the digest concentration using 0.106 g of kaolin made up to 50 mL.

## Abbreviations

The following abbreviations are used in this manuscript:

3D	Three-dimensional
ATR-FTIR	Attenuated Total Reflectance Fourier Transform Infrared
DI	Deionized
MF	Microfiltration
MIP	Mercury Intrusion Porosimetry
MP	Microplastic
PE	Polyethylene
PET	Polyethylene terephthalate
PSD	Particle size distribution
SEM	Scanning Electron Microscopy
UF	Ultrafiltration

## References

[1] Bhattacharya A., Yadav S., Kundu N., Khinvasara P., Aseri G., Jain N. (2026). Fiber to fragment: A review of microplastics from textile industry effluents, their detection, ecological impact, and sustainable microbial biodegradation strategies. Arch. Microbiol..

[2] (2023). Principles for the Analysis of Microplastics Present in the Environment.

[3] Liu M., Da X., Qiu M., Chen X., Fu K., Fan Y. (2026). Application of ceramic membranes for the removal of micro-and nanoplastics from water and wastewater: A review. Emerg. Contam. Environ. Health.

[4] Lei J., Ma Q., Ding X., Pang Y., Liu Q., Wu J., Zhang H., Zhang T. (2024). Microplastic environmental behavior and health risk assessment: A review. Environ. Chem. Lett..

[5] Wu P., Lin S., Cao G., Wu J., Jin H., Wang C., Wong M.H., Yang Z., Cai Z. (2022). Absorption, distribution, metabolism, excretion and toxicity of microplastics in the human body and health implications. J. Hazard. Mater..

[6] Leslie H.A., Van Velzen M.J., Brandsma S.H., Vethaak A.D., Garcia-Vallejo J.J., Lamoree M.H. (2022). Discovery and quantification of plastic particle pollution in human blood. Environ. Int..

[7] Law K.L., Morét-Ferguson S.E., Goodwin D.S., Zettler E.R., DeForce E., Kukulka T., Proskurowski G. (2014). Distribution of surface plastic debris in the eastern Pacific Ocean from an 11-year data set. Environ. Sci. Technol..

[8] Nihart A.J., Garcia M.A., El Hayek E., Liu R., Olewine M., Kingston J.D., Castillo E.F., Gullapalli R.R., Howard T., Bleske B. (2025). Bioaccumulation of microplastics in decedent human brains. Nat. Med..

[9] Li D., Li P., Shi Y., Sheerin E.D., Zhang Z., Yang L., Xiao L., Hill C., Gordon C., Ruether M. (2025). Stress-induced phase separation in plastics drives the release of amorphous polymer micropollutants into water. Nat. Commun..

[10] Wawer J., Panuszko A., Kozłowski D., Juniewicz J., Szymikowski J., Brodnicka E. (2025). Sustainable management of microplastic pollutions from PET bottles: Overview and mitigation strategies. Appl. Sci..

[11] Skaf D.W., Punzi V.L., Rolle J.T., Kleinberg K.A. (2020). Removal of micron-sized microplastic particles from simulated drinking water via alum coagulation. Chem. Eng. J..

[12] Kumar R., Verma A., Rakib M.R.J., Gupta P.K., Sharma P., Garg A., Girard P., Aminabhavi T.M. (2023). Adsorptive behavior of micro (nano) plastics through biochar: Co-existence, consequences, and challenges in contaminated ecosystems. Sci. Total Environ..

[13] Lin Z., Hu X., Lin H., Yu G., Shen L., Yu W., Li B., Zhao L., Ying M. (2025). Membrane technology for microplastic removal: Microplastic occurrence, challenges, and innovations of process and materials. Chem. Eng. J..

[14] Takeuchi H., Tanaka S., Koyuncu C.Z., Nakada N. (2023). Removal of microplastics in wastewater by ceramic microfiltration. J. Water Process Eng..

[15] Loh C.Y., Burrows A.D., Xie M. (2025). Sustainable polymeric membranes: Green chemistry and circular economy approaches. ACS ES T Eng..

[16] Hachemi C., Enfrin M., Rashed A.O., Jegatheesan V., Hodgson P.D., Callahan D.L., Lee J., Dumée L.F. (2023). The impact of PET microplastic fibres on PVDF ultrafiltration performance–A short-term assessment of MP fouling in simple and complex matrices. Chemosphere.

[17] Maliwan T., Hu J. (2025). Release of microplastics from polymeric ultrafiltration membrane system for drinking water treatment under different operating conditions. Water Res..

[18] Nandi B., Uppaluri R., Purkait M. (2011). Identification of optimal membrane morphological parameters during microfiltration of mosambi juice using low cost ceramic membranes. LWT-Food Sci. Technol..

[19] Hubadillah S.K., Othman M.H.D., Ismail A., Rahman M.A., Jaafar J., Iwamoto Y., Honda S., Dzahir M.I.H.M., Yusop M.Z.M. (2018). Fabrication of low cost, green silica based ceramic hollow fibre membrane prepared from waste rice husk for water filtration application. Ceram. Int..

[20] Kamgang-Syapnjeu P., Njoya D., Kamseu E., Balme S., Bechelany M., Soussan L. (2022). Bio-based ceramic membranes for bacteria removal from water. Membranes.

[21] Kolluru S.K., Raja V.K. (2026). Removal of Synthetic and Toothpaste Derived Microplastics from Wastewater Using Low-Cost Fuller’s Earth and Bagasse Ash Ceramic Membrane. Clean. Water.

[22] Issaoui M., Limousy L. (2019). Low-cost ceramic membranes: Synthesis, classifications, and applications. Comptes Rendus Chim..

[23] Dommati H., Ray S.S., Wang J.-C., Chen S.-S. (2019). A comprehensive review of recent developments in 3D printing technique for ceramic membrane fabrication for water purification. RSC Adv..

[24] Bartoletti A., Mercadelli E., Saraceni V., Sangiorgi A., Gondolini A., Melandri C., Pinasco P., Gramazio P., Fasolini A., De Maron J. (2025). 3D-printed ceramic membranes: Fabrication and hydrogen permeation performance. J. Membr. Sci..

[25] Fang Z., Sallach J., Hodson M.E. (2023). Ethanol, not water, should be used as the dispersant when measuring microplastic particle size distribution by laser diffraction. Sci. Total Environ..

[26] Mouiya M., Bouazizi A., Abourriche A., El Khessaimi Y., Benhammou A., Taha Y., Oumam M., Abouliatim Y., Smith A., Hannache H. (2019). Effect of sintering temperature on the microstructure and mechanical behavior of porous ceramics made from clay and banana peel powder. Results Mater..

[27] Sanchez-Soto P.J., García-Garzón V., Martínez-Martínez S., Pérez-Villarejo L., Sánchez-Garrido J.A., Garzón E. (2025). Influence of features and firing temperature on the ceramic properties and phase evolution of raw kaolins. Constr. Build. Mater..

[28] Bouzerara F. (2026). Comparative study on the effect of kaolin clay calcination temperature on the fabrication and properties of ceramic membranes. Int. J. Mater. Form..

[29] Njuhou S., Mouafon M., Pountouenchi A., Njindam O.R., Lecomte-Nana G.L., Njoya D. (2023). Rice husks and kaolin based ceramic membrane for filtration of slaughterhouse wastewater: Optimization study using response surface methodology (RSM) and responses interdependence analysis. Trans. Indian Ceram. Soc..

[30] Alias S.S., Harun Z., Abu Mansor S. (2020). Characterization and performance of rice husk as additive in green ceramic water filter fabricated by slip-casting. World J. Eng..

[31] Agarwalla A., Mohanty K. (2024). Fabrication and characterization of low-cost kaolin based tubular ceramic membrane for microalgal harvesting. J. Environ. Chem. Eng..

[32] Ghodrat M., Rhamdhani M.A., Brooks G., Masood S., Corder G. (2016). Techno economic analysis of electronic waste processing through black copper smelting route. J. Clean. Prod..

[33] Winter O. (1969). Preliminary economic evaluation of chemical processes at the research level. Ind. Eng. Chem..

[34] Retsch GmbH Planetary Ball Mill PM 100—Product Data Sheet. https://www.retsch.com/files/452788/product-data-sheet-pm-100.pdf.

[35] VormVrij 3D LUTUM 5M Clay Printer—Technical Datasheet. https://vormvrij.nl/download/datasheets/datasheet-LUTUM-5M.pdf.

[36] ProfiLab24 GmbH Nabertherm High Temperature Furnace with MoSi_2_ Heating Elements, Model LHT 04/17/P 580 (Item NAB_8167). https://profilab24.com/en/laboratory/laboratory-furnaces-ovens/nabertherm-high-temperature-furnace-with-mosi2-heating-elements.

[37] Sterlitech TAMI Ceramic Membrane, 0.45 Micron, 47 mm, 5/Pk (SKU 47M045). https://www.sterlitech.com/ceramic-membrane-filters-0-45-micron-47mm-5-pk-1.html.

[38] Dong Y., Wu H., Yang F., Gray S. (2022). Cost and efficiency perspectives of ceramic membranes for water treatment. Water Res..

[39] Rodrigo P.M., Sims E.M., Kommalapati R.R. (2025). Application of Hybrid Ceramic Membranes for Microplastic and Nanoplastic Separation and Improved Wastewater Treatment. Microplastics.

[40] Kim S., Hyeon Y., Rho H., Park C. (2024). Ceramic membranes as a potential high-performance alternative to microplastic filters for household washing machines. Sep. Purif. Technol..

[41] Chen X., Cao Q., Chen T., Wang D., Fan Y., Xing W. (2023). 3D printing for precision construction of ceramic membranes: Current status, challenges, and prospects. Adv. Membr..

[42] Wang D., Ling D., Lin W., Wang K., Feng Y., Liu L., Yin Y., Hu L., Huang X., Jiang G. (2024). Potential and challenges of 3D printing technology in membrane-based water treatment research. ACS ES T Water.

[43] Rodrigo-Clavero M.-E., Rodrigo-Ilarri J., Alimova K.K., Salikova N.S., Makeyeva L.A., Berdali M. (2026). Microplastics in wastewater systems of Kazakhstan and Central Asia: A critical review of analytical methods, uncertainties, and research gaps. Water.

