# Supplementary material for: Low-Cost 3D-Printed Kaolin–Rice-Husk Microporous Membranes for Microplastic Removal: A Comparative Study with a Commercial Ceramic Membrane
Source: Membranes (Basel). 2026 Oct 19;16(10):346. doi: 10.3390/membranes16100346 (PMC13706905; doi:10.3390/membranes16100346)
Supplement: Supplementary file 1 [file membranes-16-00346-s001.zip › membranes-4613168-supplementary.pdf]

Supplementary Material

Cost estimation of the 3D-printed rice husk/kaolin ceramic membrane

This section provides the detailed calculations underlying the cost assessment presented in Section 3.x of the main text. All values refer to a single fabrication run of 20 discs, each 40 mm in diameter with an effective filtration area of 12.57×10^{-4} m^2. Monetary amounts are expressed in United States dollars at a conversion rate of 447 KZT USD^{-1}, and electricity was charged at the Astana municipal tariff of 28.12 KZT kWh^{-1} inclusive of value added tax, equivalent to 0.0629 USD kWh^{-1}.

Table S1. Electrical energy consumed per fabrication run of 20 membranes.

Equipment	Rated power (kW)	Operating time (h)	Energy (kWh)	Cost (USD)
Retsch PM 100 planetary ball mill	1.25	12	15.0	0.94
VormVrij LUTUM 5M clay printer	0.35	2	0.7	0.04
Nabertherm LHT 04/17 furnace	3.60	24	86.4	5.43
Drying oven (rice husk) [a]	1.50	0.2	0.3	0.02
**Total**	—	—	**102.4**	**6.44**

[a] The entire annual rice husk requirement (0.6 kg, one 5 L bag) was washed and dried in a single 2 h operation at 90 °C; the associated energy was apportioned across the ten runs carried out per year, equivalent to 0.2 h per run.

Table S2. Equipment acquisition values, annual depreciation and allocation per fabrication run.

Equipment	V_0, 2021 (USD)	Annual depreciation (USD yr^{-1})	Hours per run	Cost per run (USD)
Retsch PM 100	9,877	889	12	5.33
VormVrij LUTUM 5M	8,148	733	2	0.73
Nabertherm LHT 04/17	13,713	1,234	24	14.81
Drying oven	2,058	185	0.2	0.02
**Total**	**33,796**	**3,041**	**38.2**	**20.89**

Purchase prices were estimated from current supplier listings, adjusted back to 2021 using a cumulative inflation factor of 1.215. Depreciation was calculated by the straight-line method over a 10-year life with a residual value of 10%. Since the equipment is shared with other work, depreciation was charged in proportion to the hours each instrument is used per run, assuming 2000 available hours per year. Repairs and servicing were taken as 2% of equipment value per year and charged on the same basis, giving 4.64 USD per run.

Table S3. Breakdown of the total fabrication cost.

**Cost head**	**Cost per run (USD)**	**Cost per membrane (USD)**	**Share of total (%)**
Raw materials	0.39	0.0195	0.7
Electricity	6.44	0.322	10.8
Labour	22.73	1.136	38.1
Laboratory expenses	4.55	0.227	7.6
Depreciation	20.90	1.045	35.0
Repairs and maintenance	4.64	0.232	7.8
**Total**	**59.64**	**2.98**	**100**

Table S4. Comparison with the commercial ceramic membrane used as a benchmark.

**Parameter**	**This study**	**TAMI commercial membrane**
Composition	Rice husk / kaolin (1:4)	TiO_2 on ceramic support
Disc diameter (mm)	40	47
Effective area (× 10^{-4} m^2)	12.57	17.35
Cost per membrane (USD)	2.98	54.45
Cost per unit area (USD m^{-2})	2,373	31,383

The commercial membrane is supplied in packs of five at 272.24 USD, equivalent to 54.45 USD per disc. On this basis the membrane developed in the present work is cheaper by a factor of 18.3 per disc and 13.2 per unit of filtration area.

Table S5. Fabrication process parameters

**Parameter**	**Value**
Kaolin : rice husk mass ratio	4: 1
Rice husk drying	90 °C, 2 h
Ball milling	PM 100 (Retsch), 100 rpm, 6 cycles × 6 hrs
Paste water content	approx. 100 g of water per batch
3D printer / method	LUTUM, extrusion-based
Nozzle diameter	4 mm
Layer height	3 mm
Printing speed	0.1 mm s^{-1}
Green disc dimensions	45 mm diameter × 3 mm thickness
Green-body drying	ambient temperature
Sintering temperature	1200 °C / 1500 °C

Parameter	Value
Heating (ramp) rate	5 °C min^{-1}
Dwell time at sintering temperature	24 hrs
Cooling	to room temperature over 24 h
Linear shrinkage	11.1% (45 mm green body vs 40 mm fired)
Yield	20 usable discs

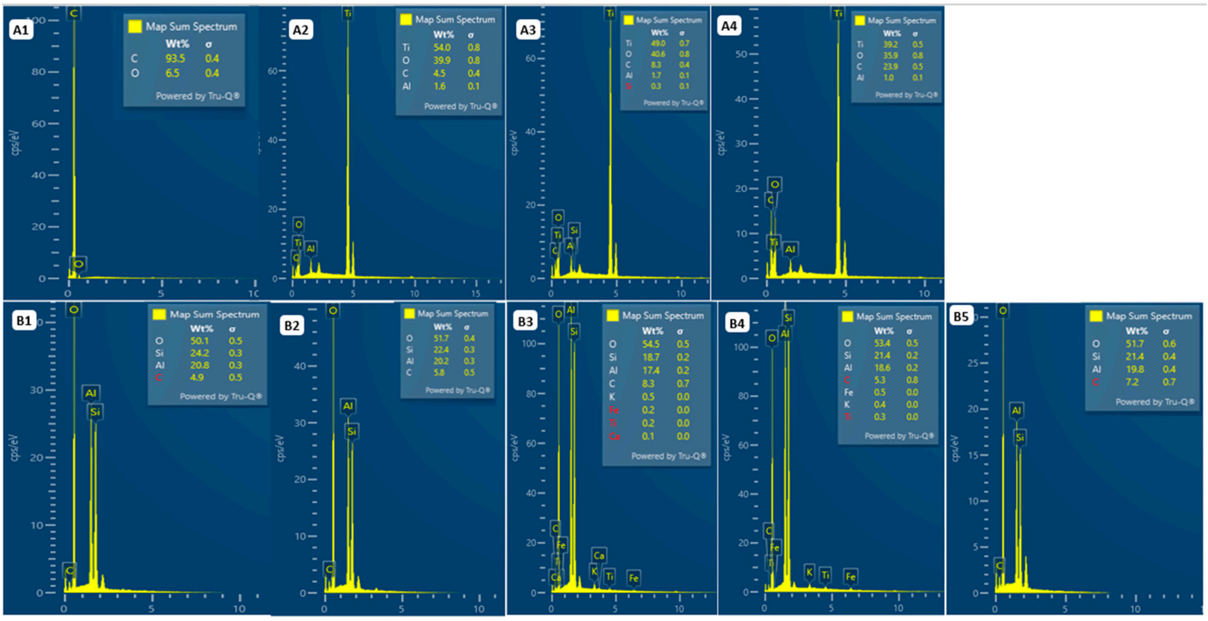

Figure S1. EDS map sum spectra and elemental compositions (wt%) of the TAMI commercial membrane and the 3D-printed rice husk/kaolin membrane, before and after filtration of PE and PET microplastics of different size fractions.
A1:TAMI 1-10 PE; A2:TAMI 600-710 PE; A3: TAMI 75-150 PET; A4: TAMI 10-45 PET; B1: 3D 1-10 PE; B2: 3D 600-710 PE; B3: 3D 75-150 PET; B4: 3D 10-45 PET; B5: Virgin 3D

Table S6. Elemental composition of the kaolin precursor determined by ICP-OES after acid digestion (HNO_3 5 mL, H_2SO_4 1 mL, $HClO_4$ 1 mL, HF 2 mL; neutralized with 5 mL boric acid), reported as blank-corrected means (n = 3). Solid-phase content was obtained from the digest concentration using 0.106 g of kaolin made up to 50 mL.

Element	Digest concentration (mg L^{-1})	Content in kaolin (mg kg^{-1})
Si	155.7	73500
Al	52.5	24800
Ti	9.52	4490
Fe	8.54	4030
K	4.53	2140
P	1.93	909
Na	0.54	255
Ba	0.43	202

Element	Digest concentration (mg L^{-1})	Content in kaolin (mg kg^{-1})
Ca	0.28	134
Sr	0.27	126
Pb	0.19	89
V	0.16	74
Mg	0.12	56
Zn	0.06	28
Cu	0.05	25
Cr	0.05	25
Mn	0.03	15
Ni	0.016	8
Co	0.015	7
Li	0.013	6
As	0.004	1.9
Mo	0.003	1.4
Hg	0.002	0.9
Cd	0.001	0.5
Se	< DL	< DL
